# OB Nest randomized controlled trial: a cost comparison of reduced visit compared to traditional prenatal care

**DOI:** 10.1186/s12884-021-03557-3

**Published:** 2021-01-21

**Authors:** Regan N. Theiler, Yvonne Butler-Tobah, Matthew A. Hathcock, Abimbola Famuyide

**Affiliations:** 1 Department of Obstetrics and Gynecology, Mayo Clinic, 200 First Street SW, Rochester, MN 55905 USA; 2 Biomedical Statistics and Informatics, Mayo Clinic, Rochester, MN 55905 USA

**Keywords:** Pregnancy, Prenatal, Obstetrics, Midwifery, Obstetrician, Nest, Telemedicine

## Abstract

**Background:**

Traditional prenatal care includes up to 13 in person office visits, and the cost of this care is not well-described. Alternative models are being explored to better meet the needs of patients and providers. OB Nest is a telemedicine-enhanced program with a reduced frequency of in-person prenatal visits. The cost implications of connected care services added to prenatal care packages are unclear.

**Methods:**

Using data from the OB Nest randomized, controlled trial we analyzed the provider and staff time associated with prenatal care in the traditional and OB Nest models. Fewer visits were required for OB Nest, but given the compensatory increase in connected care activity and supplies, the actual cost difference is not known. Nursing and provider staff time was prospectively recorded for all patients enrolled in the OB Nest clinical trial. Published 2015 national wages for healthcare workers were used to calculate the actual labor cost of providing either traditional or OB Nest prenatal care in 2015 US dollars. Overhead expenses and opportunity costs were not considered.

**Results:**

Total provider cost was decreased caring for the OB Nest participants, but nursing cost was increased. OB Nest care required an average of 160.8 (+/− 45.0) minutes provider time and 237 (+/− 25.1) minutes nursing time, compared to 215.0 (+/− 71.6) and 99.6 (+/− 29.7) minutes for traditional prenatal care (*P* < 0.01). This translated into decreased provider cost and increased nursing cost (P < 0.01). Supply costs increased, travel costs declined, and overhead costs declined in the OB Nest model.

**Conclusions:**

In this trial, labor cost for OB Nest prenatal care was 34% higher than for traditional prenatal care. The increased cost is largely attributable to additional nursing connected care time, and in some practice settings may be offset by decreased overhead costs and increased provider billing opportunities. Future efforts will be focused on development of digital solutions for some routine nursing tasks to decrease the overall cost of the model.

**Trial registrations:**

ClinicalTrials.gov Identifier: NCT02082275.

## Introduction

The COVID-19 pandemic has caused a rapid reassessment of prenatal care models, with adoption of remote visits and decreased visit models expanding rapidly over the past year [1]. The largely traditional model of prenatal care in the United States is expensive, resource-intensive, and fraught with nationwide variation. If uniformly applied to the approximately 4 million pregnancies in the United States each year, the clinic personnel, administrative infrastructure, office space, and miscellaneous cost of providing care is clearly substantial [2]. In 2008, the national hospital bill was approximately 1.2 trillion dollars, with pregnancy and delivery accounting for the most expensive condition treated in the United States [3]. A recent analysis of obstetric care suggests implementing a reduced prenatal visit schedule for appropriate expectant mothers could reduce the cost of prenatal care by 2.5–13%, depending on the intensity of supplemental remote care [2]. However, quality studies investigating the costs of supplemental remote care in the setting of reduced prenatal care models remain limited.

In March 2014, we developed and studied an alternative bundle of prenatal care – Mayo Clinic OB Nest - which included fewer on-site clinic appointments supplemented with virtual visits with an assigned nurse, home monitoring devices (fetal heart rate and blood pressure devices) and access to an online prenatal community of expectant mothers [4–6]. We found that OB Nest improved patients’ satisfaction with care, decreased prenatal –related stress, and maintained quality of care [6]. However, the amount of time obstetric nurses spent with OB Nest patients was significantly higher than expected [6].

The Mayo Clinic OB Nest program has demonstrated improved patient satisfaction compared to routine prenatal care, while maintaining excellent maternal and neonatal outcomes. We hypothesized that this abbreviated prenatal visit schedule supplemented with connected care, in addition to improving patient experience, would decrease the cost of prenatal care delivery. Results are increasingly relevant as we witness the rapid, large-scale redesign of prenatal care delivery during the current pandemic.

## Materials and methods

Between March 2014 and January 2015, we conducted a single center randomized controlled trial within the Outpatient Obstetrics Division at Mayo Clinic, a tertiary care academic center in Rochester, Minnesota [4]. Enrollment criteria included expectant mothers between 18 and 36 years old, < 13 weeks gestation, who had their pregnancy documented as low risk by an obstetrician and had the ability to provide informed consent. Exclusion criteria were described elsewhere [6, 7]. This study was reviewed and approved by the Mayo Clinic Institutional Review Board (Reference #13–009513) and registered at ClinicalTrials.gov (Identifier: NCT02082275). 300 patients were included in the OB Nest trial. Of these 300, a total of 39 patients were ultimate excluded from the trial (12 patients because of study withdrawal, 6 due to miscarriages, 5 who transferred care to another provider, and 16 who developed high-risk pregnancy conditions). The remaining enrolled patients include 130 in the traditional care arm and 131 in the OB Nest arm of the trial, for whom 3440 individual appointments were included in this analysis.

Low risk obstetric patients scheduled for routine prenatal were seen by a mixture of Certified Nurse Midwives (CNM) and OB physicians. Participants randomized to OB Nest were assigned to: (i) Eight planned clinic appointments with a clinician (physician or midwife), (ii) Six planned virtual (phone or online) connected care visits with a Registered Nurse (RN) dedicated to OB, (iii) home digital sphygmomanometer and handheld fetal Doppler, and (iv) access to an online prenatal care community designated for OB Nest participants. Participants, nurses, or clinicians could at any point request further appointments if deemed clinically necessary. Participants in the usual prenatal care model were scheduled for the traditional (up to 13) clinic appointments with their clinician per ACOG recommendations.

We measured healthcare utilization by extracting number of office visits, in-person visit time, nursing visit time (both remote and in person), and nursing care coordination time. Data was captured during the trial using Mayo Clinic’s Workload Measurement and Reporting System. Hourly salary data for health professionals in 2015 was obtained from the Department of Labor, and fringe benefits were estimated at 36% based on standard federal rates [8–10]. Thus, labor costs were calculated using an hourly MD rate of $115.26, CNM of $52.42 and RN of $37.28, with a standard fringe rate. Supply costs were calculated based on actual cost of purchasing home fetal dopplers and sphygmomanometers. In addition to patient education, nurse connected care time included: care coordination, management of labs and tests, assessment of symptoms and medical history, and management of prescriptions and prior authorizations. Travel distance and time were estimated by calculating the distance from the patients’ home zip codes at enrollment to the clinic site, multiplying by the number of on-site appointments. Driving costs were estimated using standard IRS mileage reimbursement rates for 2015 [11]. Parking costs were not captured for this study.

### Statistical methods

Continuous variables are described using means and standard deviations, with comparison using a two-sample t-test. Categorical variables are summarized as count and percent, with analysis by chi-squared test. Appointments were classified as per protocol if they corresponded to the planned gestational age of scheduled protocol visits. Summaries of patient appointment data were done using two sample t-tests and chi-square tests as appropriate. Non-parametric methods were considered, but their use did not result in significant changes to the study outcomes. Statistical analysis was performed using SAS version 9.4.

## Results

From March 2014 through January 2015, 1, 515 expectant mothers were screened for eligibility and 300 mothers < 13 weeks gestation were recruited and randomized into OB Nest or usual care with 150 in each arm. A total of 267 patients completed the trial. Basic demographics of the study population are shown in Table 1. As previously described, OB Nest saved an average of 2.8 obstetric provider appointments per patient while significantly improving the patient experience [6]. Healthcare personnel time spent in provision of routine prenatal care is shown in Table 2. Intrapartum and postpartum care effort is not included, and data represent actual time spent in a blended physician/midwifery practice model per single low-risk pregnancy episode. Visit protocol compliance did not differ significantly between the two groups. Comparison of OB Nest care to traditional prenatal care shows an average savings of 54 min (160.8 vs 215, *P* < 0.01) provider time spent per pregnancy for OB Nest. In comparison, a total of 237 min increase in RN effort, including an average of 174 (232.7 vs 58.9, P < 0.01) additional minutes of nurse connected care contact per pregnancy was spent on OB Nest care.

**Table 1:** Summary of patient demographics in OB Nest and traditional care arms

Variable	OB Nest (***n*** = 131)	Usual Care (***n*** = 130)	***P***-Value
Maternal Age, mean ± SD	29.6 (3.1)	29.9 (3.6)	0.45^1^
Maternal Age ≥ 35, n (%)	6 (4.6%)	8 (6.2%)	0.57^2^
Caucasian race, n (%)	118 (90.1%)	117 (90.0%)	0.98^2^
Body mass index, mean ± SD	25.2 (5.3)	25.8 (6.8)	0.97^1^
Body mass index > 30, n (%)	22 (16.8%)	23 (17.7%)	0.85^2^
Gestational Age at Delivery (weeks)	39.8 (1.2)	39.7 (1.8)	0.77^1^
Gestational Age < 37 Weeks	4 (3.1%)	3 (2.3%)	> 0.99^3^
Gestational age at first appointment	8.7 (1.4)	8.6 (1.3)	0.74^1^
Gravida of 1, n (%)	41 (31.3%)	45 (34.6%)	0.57^2^
Parity, n (%)			0.51^2^
0	53 (40.5%)	57 (43.8%)	
1	47 (35.9%)	38 (29.2%)	
2+	31 (23.7%)	35 (26.9%)	
Past Cesarean Delivery, n (%)	8 (6.1%)	14 (10.8%)	0.18^2^
Married/marriage-like relationship, n (%)	117 (89.3%)	115 (88.5%)	0.56^2^
Education, n (%)			> 0.84^3^
High School graduate or less	3 (2.3%)	5 (4.0%)	
Some college or associates degree	31 (24.2%)	27 (21.6%)	
Four-year college graduate	55 (43.0%)	52 (41.6%)	
Graduate/ professional school degree	39 (30.5%)	41 (32.8%)	
*Missing*	3	5	
Private Insurance, n (%) Missing	120 (91.6%)	112 (86.2%)	0.16^2^
Annual Household Income, n (%)			0.80^2^
< $40,000	16 (12.6%)	13 (10.5%)	
$40,000 to $79,999	38 (29.9%)	41 (33.1%)	
≥ $80,000	73 (57.5%)	70 (56.5%)	
*Missing*	4	6	
Driving Distance^**^	3.0 (1.0, 12.3)	2.6 (1.0, 13.0)	0.49^4^

^1^T-Test ^2^Chi-Square ^3^Fisher Exact Test ^4^Wilcoxon Rank sum

**Table 2:** Overview of appointment time and personnel costs for team prenatal care

Description	OB Nest (n = 131)	Usual Care (n = 130)	P-Value
In Person Visits, n	9.9 (3.3)	13.7 (3.2)	< 0.01
Provider Visits, n	6.3 (1.7)	9.4 (1.9)	< 0.01
Nurse Encounters*, n	22.3 (7.4)	5.5 (3.7)	< 0.01
Other Visits**, n	2.2 (1.8)	2.3 (1.6)	0.61
Provider Time, min	160.8 (45.0)	215.0 (71.6)	< 0.01
Nurse Time*, min	237 (25.1)	99.6 (29.7)	< 0.01
Other Time**, min	82.3 (54.2)	86.4 (51.3)	0.41
Provider Cost, 2015 U.S. Dollars			
Physician	$154 (96)	$114 (145)	< 0.01
Midwife	$70 (42)	$136 (87)	< 0.01
Nurse Cost	$252 (85)	$106 (52)	< 0.01
Total Personnel Cost	$476 (120)	$356 (129)	< 0.01

*Includes connected care visits and in person visits

**Other visits or time refers to ultrasound, laboratory, and genetic counseling visits

The balance of decreased provider time and increased nursing time resulted in a net shift of expense to the nursing staff, with an overall average increase of $120 in personnel cost for OB Nest prenatal care in comparison to traditional prenatal care (*P* < 0.01). In addition to increased staff expense, the OB Nest model increased supply costs by approximately $101 per patient for purchase of home blood pressure and fetal 8oppler devices. Most patients did have access to a personal or shared scale, as well as internet access and these costs were not included in our overall analysis. Assuming that clinic overhead costs decrease in proportion to the 35% decrease in physical patient visits (from 13.7 to 9.9, *P* < 0.01), the cost of clinic overhead was also decreased from an estimated 53 to 34% of expected revenue [12]. The actual dollar impact of this overhead decrease varies based on practice location and payer mix, but in most cases will offset the increase in supply costs. For this reason, and because of practice-specific overhead costs, supply and overhead changes were not included in the final calculation of cost difference. In our study, we did find a significant difference in the distance driven for appointments by group (Nest 29.6 miles vs. traditional 36.4 miles, *P* < 0.01), but the cost of driving was not significantly different (Table 4). We did not capture parking costs, which may also differ by model and contribute to the overall cost of care.

Finally, we predict that among the low-risk prenatal care models we examined, the least expensive is midwifery care in the traditional model **(**Table 3**).** The data for this trial were gathered using a combination of midwife and physician care. Assuming that physicians and certified nurse midwives spend the same amount of time per prenatal patient, we calculated the predicted cost (provider time x cost/hr. + nursing cost) of midwifery-only and physician-only provider models. This comparison suggests that a traditional midwifery model of care costs less than either OB Nest care or traditional physician care.

**Table 3:** Predicted personnel cost of provider models, in 2015 U.S. Dollars

Cost by provider model	OB Nest, mean (SD)	Usual care, mean (SD)	P
Midwife-only Care	$392 (94)	$294 (94)	< 0.01
Physician-only Care	$560 (122)	$519 (163)	0.02

**Table 4:** Transportation

Drive summaries--Median (IQR)			
Driving Distance (Miles)^**^	3.0 (1.0, 12.3)	2.6 (1.0, 13.0)	0.49^4^
Driving Costs (USD)	**2.8 (1.0, 94)**	**3.5 (1.3, 16.1)**	**0.032**
Total Miles Driven	**29.6 (11, 98.4)**	**36.4 (14, 169)**	**0.032**

**Drive distance is estimated as distance between Mayo Clinic Rochester zip code 55905 and the patient’s zip code at time of enrollment

## Discussion

In the setting of the OB Nest randomized controlled trial, the OB Nest model shifted some work of prenatal care to the nursing workforce, opening up approximately one additional hour of provider time for every prenatal care episode. For a practice averaging 150 deliveries per provider per year, this translates to an opportunity for 135 additional patient care hours in clinic per provider annually. In this analysis, we did not estimate the revenue opportunity associated with that increased provider time. Depending on the practice setting, payer mix, and overhead costs, increased provider revenue may offset the additional cost incurred for nursing time. Thus, for practices with provider shortages, high overhead, and adequate nursing support, OB Nest may be cost saving in its current form.

We previously reported significantly improved patient experience scores with OB Nest compared to traditional prenatal care. Given the continual pursuit of greater value in healthcare, we must weigh the better patient experience against the slightly increased cost of the OB Nest model. In settings with acute provider shortages, for instance, the use of additional nurse time for the OB Nest model may allow a practice to care adequately for more prenatal patients than the traditional model would otherwise afford.

With a current and worsening Ob/Gyn physician shortage [13, 14], moving to prenatal care models that require less physician time will allow more patients to benefit from adequate care. Given the important trade-off of increased patient satisfaction with OB Nest care, some practices in competitive markets may also find OB Nest makes financial sense by attracting patients to a practice. Finally, practices with physician providers may find the model to be more cost-effective than those with a predominant midwifery staffing model, as the cost difference between OB Nest and traditional physician care is only $41 (USD). Additional patient-specific considerations may include the relative value of patient time lost from work, as well as transportation costs for in-person clinic visits. Pandemic-specific benefits of the OB Nest model include the ability to provide quality prenatal care with significant decrease in personal contact.

### Strengths and limitations

The analyzed data were obtained prospectively in a patient-specific fashion using a real-time workload monitoring system in the context of the OB Nest randomized controlled trial, and the accuracy of the information is a strength of the study. Limitations of the study include the practice setting in a tertiary referral center, and exclusion of postpartum and intrapartum care costs from the study. The findings thus may not be generalizable to other settings, and the impact on global OB package costs is not known. We were also limited in our ability to measure practice overhead expenses, and individual practices may not have the same results with OB Nest implementation. Finally, the ability to calculate cost effectiveness in terms of dollars saved per quality-adjusted life year is limited by the lack of baseline cost effectiveness data for prenatal care in general.

Future practice optimizations and digital connectivity solutions will allow transition of more routine aspects of nursing care, such as recording blood pressure and blood glucose values and conveying gestational-age specific patient education, to the automated digital realm. We anticipate that with appropriate automation, the personnel costs will ultimately decrease, allowing the OB Nest model to become more cost effective in future iterations.

## Data Availability

The datasets generated and/or analysed during the current study are not publicly available due to private funding and presence of personal identifiers, but may be available from the corresponding author on reasonable request.
